# Colistin-Resistant *Acinetobacter Baumannii* Bacteremia: A Serious Threat for Critically Ill Patients

**DOI:** 10.3390/microorganisms8020287

**Published:** 2020-02-20

**Authors:** Georgios Papathanakos, Ioannis Andrianopoulos, Athanasios Papathanasiou, Efthalia Priavali, Despoina Koulenti, Vasilios Koulouras

**Affiliations:** 1 University Hospital of Ioannina, Intensive Care Unit, Stavros Niarchos Avenue, 45500 Ioannina, Greece; jandri0@yahoo.gr (I.A.); thanasis.papathanasiou@gmail.com (A.P.); vpkoulouras@yahoo.gr (V.K.); 2 Department of Microbiology, Medical School, University of Ioannina, 45110, Ioannina, Greece; 3 Burns, Trauma and Critical Care Research Centre, UQ Centre for Clinical Research (UQCCR), Faculty of Medicine, The University of Queensland, Brisbane, Herston Campus, Brisbane QLD 4029, Australia; d.koulenti@uq.edu.au; 4 2nd Critical Care Department, Attikon University Hospital, Rimini Street, 12463, Athens, Greece

**Keywords:** *acinetobacter baumannii*, XDR, colistin resistance, sepsis, fulminant septic shock, mortality, intensive care unit, blood stream infection, bacteremia

## Abstract

The prevalence of *acinetobacter baumannii* (AB) as a cause of hospital infections has been rising. Unfortunately, emerging colistin resistance limits therapeutic options and affects the outcome. The aim of the study was to confirm our clinically-driven hypothesis that intensive care unit (ICU) patients with AB resistant-to-colistin (ABCoR) bloodstream infection (BSI) develop fulminant septic shock and die. We conducted a 28-month retrospective observational study including all patients developing AB infection on ICU admission or during ICU stay. From 622 screened patients, 31 patients with BSI sepsis were identified. Thirteen (41.9%) patients had ABCoR BSI and 18/31 (58.1%) had colistin-susceptible (ABCoS) BSI. All ABCoR BSI patients died; of them, 69% (9/13) presented with fulminant septic shock and died within the first 3 days from its onset. ABCoR BSI patients compared to ABCoS BSI patients had higher mortality (100% vs. 50%, respectively (*p* = 0.001)), died sooner (*p* = 0.006), had lower pH (*p* = 0.004) and higher lactate on ICU admission (*p* = 0.0001), and had higher APACHE II (*p* = 0.01) and Charlson Comorbidity Index scores (*p* = 0.044). In conclusion, we documented that critically ill patients with ABCoR BSI exhibit fulminant septic shock with excessive mortality. Our results highlight the emerging clinical problem of AB colistin resistance among ICU patients.

## 1. Introduction

*Acinetobacter baumannii* (AB) is an opportunistic gram-negative with a tendency to colonize the hospital environment: bed rails, floors, ventilator pads, supply carts and infusion pumps in the Intensive Care Unit (ICU) [1,2]. It has the remarkable capacities to survive in dry conditions for a long period of time, to be resistant to disinfectants and form biofilm on abiotic surfaces [3], and also to develop resistance to antibiotics. It predominantly infects critically ill hospitalized patients and it is a frequent cause of nosocomial infections, mainly ventilator-associated pneumonia and bloodstream infection [4].

Although once thought to be benign and previously regarded as an organism of questionable pathogenicity, over the past several years AB has been shown to have increasing resistance to antibiotics affecting both empirical and guided treatment [3]. Today, most AB strains isolated in ICUs are not only resistant to b-lactams and fluroquinolones, but also to carbapenems, aminoglycosides, and possibly to all standard antimicrobial agents, except colistin or tigecycline [5,6,7,8,9,10]. The clinical manifestations of bacteremia of this strain of AB, known as extensively drug-resistant (XDR AB), ranges from benign transient bacteremia to fulminant septic shock [11]. XDR AB bacteremia is accompanied with excess mortality [12,13], excess length of ICU hospitalization, prolonged hospital stay [14], and higher overall costs [15,16]. XDR AB has been reported worldwide as a major cause of healthcare-associated infections and nosocomial outbreaks [17], and is now recognized as one of the most difficult hospital pathogens to be treated and controlled and is considered a global threat in the health-care setting [18].

This increasing worldwide prevalence of AB drug resistance has made colistin an important therapeutic option for XDR AB. Unfortunately, resistance to colistin has been reported among AB clinical strains [19,20,21,22,23,24].

Despite the potential magnitude of the problem, data regarding the clinical, microbiological, and molecular characteristics of ABCoR infections remain very limited [25]. Most studies include patients hospitalized in several wards and not especially in an ICU [25,26,27], while in a recent prospective observational study mortality rate for patients with ABCoR infection in an ICU setting was found to be as high as 85% [28].

Over the last year, a colistin resistant XDR AB strain has emerged in our ICU. Our clinically based observation suggested that patients with bloodstream infection (BSI) of this strain have a high mortality by developing a fulminant course of septic shock. The aim of our study was to confirm the above-mentioned observation by investigating the impact of XDR AB BSIs on the mortality of ICU patients and to compare the characteristics of ABCoR BSIs with the colistin-sensitive (ABCoS) ones.

## 2. Materials and Methods

The study was conducted in accordance with the Declaration of Helsinki, and the protocol was approved by the Ethics Committee of the University Hospital of Ioannina (reference number 1092). Informed consent was waived because of the observational retrospective nature of the study.

This is a retrospective observational study of 28 months duration conducted in a 14-bed ICU of a university hospital. During the study period (June 2017 to October 2019), all cultures obtained from the ICU patients were screened and all patients that were admitted to ICU with or developed AB infection were included in the study.

For every patient with established AB infection demographic characteristics, medical history, cause of ICU admission, and APACHE II score were recorded [29]. Sequential (sepsis-related) organ failure assessment (SOFA) score was calculated at ICU admission, 24 h before and on the day that infection was documented (infection onset) [30]. Patients with cancer under treatment, on chronic corticosteroid treatment, with autoimmune disease and/or immunosuppressant treatment, cirrhosis, and end-stage renal disease, were considered to be patients with an impaired immune response. For patients with positive blood cultures, Charlson Comorbidity Index score was calculated as well [31]. During the study period, for all patients with AB infection, we recorded baseline biochemical parameters, risk factors, empirical antibiotic therapy, days from hospital admission to sepsis, days from ICU admission to sepsis, days from admission to positive blood cultures, days from hospital admission to shock, and time from sepsis to shock development. Clinical complications were recorded as well and defined according to international guidelines. These included the development of acute respiratory distress syndrome (ARDS), acute kidney injury (AKI) [32], need for renal replacement therapy (RRT), septic myocardiopathy, thrombocytopenia (count of platelets less than 100,000/μL), or liver dysfunction [33]. For all documented infection by AB, antibiograms were also obtained and analyzed to identify the susceptibility of AB to the antibiotics.

Susceptibility of antibiotics to AB was assessed by VITEK 2 system and susceptibility to colistin was assessed by broth microdilution with resistant defined as having a colistin minimum inhibitory concentration (MIC) breakpoint >2 mg/L. Vitek 2 system (bioMerieux, Marcy I ’Etoile, France) is a fully automated system utilizing a fluorogenic methodology for organism identification and a turbidimetric method for susceptibility testing, using a 64 well card that is barcoded with information on card type, expiration date, lot number, and unique card identification number. VITEK 2 antimicrobial susceptibility testing has shown to have a high degree of agreement with standard methods for determining the MIC of antimicrobials, with a gain-of-time of hours to days and improved reproducibility [34,35]. XDR AB were defined as those isolates being non-susceptible to at least one agent in all but two or fewer antibiotic classes. Appropriate treatment was defined by the utilization of at least one susceptible antibiotic against AB during the infection period.

Definitions: Definitions of sepsis and septic shock were based on recent SEPSIS-3 international guidelines [36]. Sepsis is a clinical syndrome of life-threatening organ dysfunction caused by dysregulated host response to infection and defined as suspected or documented infection and an acute increase of ≥2 SOFA points. Septic shock was identified when a clinical construct of sepsis with persisting hypotension, requiring vasopressor therapy to elevate mean arterial pressure ≥65 mm Hg and lactate >2 mmol/L despite adequate fluid resuscitation was present [36]. AB infection was defined as the clinical manifestation of infection, which could be microbiologically confirmed by the isolation of the specific pathogen in cultured material. Ventilator associated pneumonia was defined as pneumonia occurring later than 48 h post endotracheal intubation [37].

Mortality: Sepsis related deaths were categorized as deaths directly linked to AB infection and its implications (sepsis, septic shock, and multiple organ dysfunction syndrome-MODS—due to septic shock).

Statistical analysis: Numerical variables were presented as the mean ± standard deviation, or median, min-max values when appropriate. Comparisons of continuous variables were performed by Mann-Whitney test for non-normally distributed variables, whereas w^2^ tests were used for categorical variables. The assumption concerning the proportionality of hazards was graphically assessed and met for all covariates of Kaplan-Meier survival plots of matched groups were constructed and compared by using the log-rank test. Significance was defined as a *p*-value < 0.05 in all cases. SPSS^®^ 21.0 (Chicago, IL, USA) was used for all analyses.

## 3. Results

**Figure 1:**
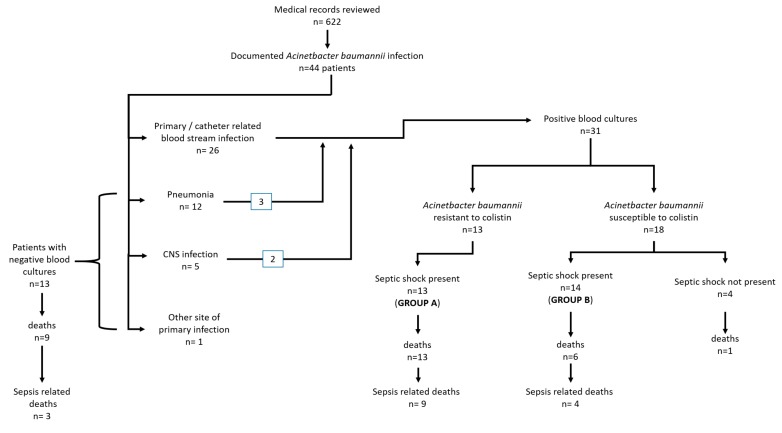
Study population flowchart. CNS: Central Nervous System.

During the study period, 44 (mean age 65.3 ± 17.6 years) out of a total of 622 patients were identified with infection by AΒ. The flowchart of the study population is shown in Figure 1.

### 3.1. Patient Characteristics

The baseline characteristics of these patients and causes of ICU admission are shown in Table 1, while AΒ infection characteristics are shown in Table 2. Out of 44 patients, 39 (88.6%) developed AB sepsis. Of these, in 28/39 (71,8%) it occurred during ICU hospitalization, while in the other 11/39 (28.2%) patients, AB sepsis itself was the cause of ICU admission. Twenty-nine patients (65.9%) fulfilled the criteria for septic shock (Table 2).

Twenty-one out of 44 infections were due to a colistin-resistant AΒ strain. Six of the above AB infections were pan-resistant (PDR), featuring intermediate sensitivity to tigecycline only. Mortality was high in this group, as 18/21 (85.7%) died during their admission. Data on previous antibiotic usage were available in 18/21 of these patients. Only 6/18 (33%) of these patients had at some point prior to their infection received colistin.

In addition, 15/40 (37.5%) patients that developed sepsis died in less than 72 h, featuring septic shock and MODS. This was irrespective of appropriate antibiotic treatment and almost all of these patients had a high SOFA score 24 h before the onset of sepsis (10.5 ± 3.8 h), indicating that there was already significant organ dysfunction in place.

### 3.2. Patients with Positive Blood Cultures Due to AΒ

Among the 44 patients, 31 AΒ were identified with sepsis due to BSI by (31/44, 70.5%). Of these, 26 were primary- or catheter-related BSI, and in the other five positive blood cultures were associated with pneumonia (three patients) and central nervous system infections (two patients) (Figure 1).

Between the 31 septic patients with positive blood cultures, AΒ was colistin-resistant in 13 patients. All 13 patients developed septic shock. In the other 18 patients, AΒ was susceptible to colistin. In these 18 patients, septic shock was evident in 14 out of 18 (78%). Univariate analysis showed that patients with AB resistant to colistin tend to be older, with higher APACHE II and Charlson Comorbidity Index scores, lower levels of pH and higher level of lactate on admission and maximum levels of lactate during hospitalization (Table 3). Additionally, the duration of shock and the duration from the onset of sepsis to death were statistically significant between patients with AB resistant to colistin and AB susceptible to colistin (Table 3). Multivariate analysis did not reveal any statistical significance for any of the above-mentioned parameters probably due to the small number of cases.

### 3.3. Patients’ Outcome

A total of 29 deaths were recorded (mortality of all causes 29/44, 65.9%). Of these, 16 deaths (16/29, 55.1% of all deaths) were directly related to sepsis from AB (sepsis related mortality 16/44, 36.4%).

Patients with BSI by AB and septic shock (*n* = 27) were further divided into two groups based on the susceptibility of AB to colistin: Group A, patients infected by ABCoR (*n* = 13) and group B, patients infected by ABCoS (*n* = 14).

**Figure 2:**
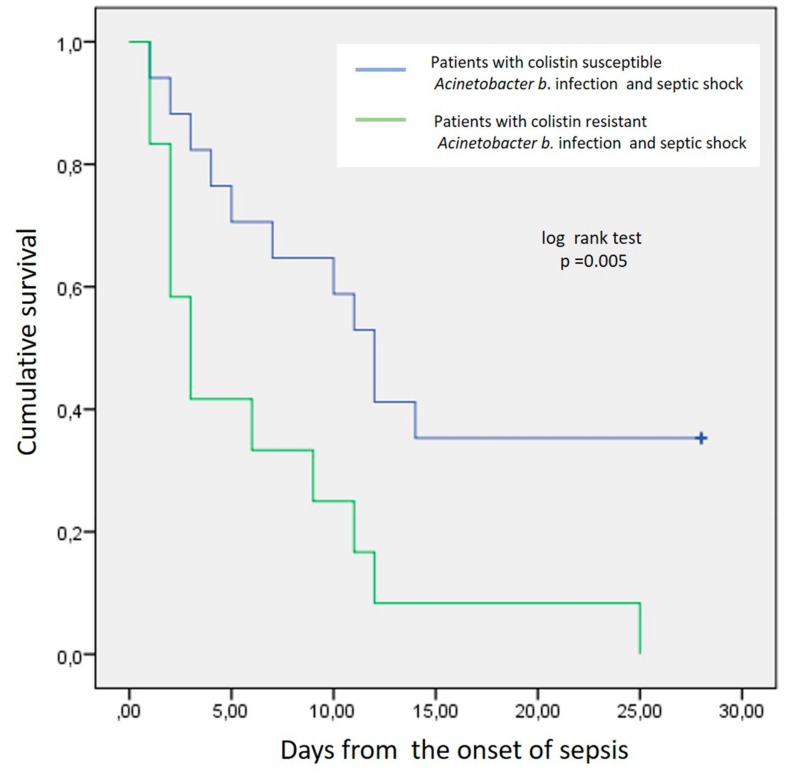
Kaplan-Meier survival curve for total mortality of AB septic shock patients.

Septic shock was documented in all patients of group A within the first hours of the onset of sepsis. All group A patients died (13/13, 100%, sepsis related deaths 9/13, 70%). In group B, patients’ mortality rate was significantly lower compared to group A (6/14, 42.8%, sepsis related deaths 4/6, 66.6%, *p* = 0.026). Time from the onset of sepsis till death for group A patients was shorter compared to group B patients (3 days—min 1 to 9 days and 6 days—min 3 to 25 days), (*p* = 0.005). The estimated 28-day Kaplan-Meier survival curve for total mortality is shown in Figure 2. The Kaplan-Meier survival curve for total mortality diverged at the 3rd day, suggesting that the significant difference in mortality is evident within the first three days from the onset of septic shock.

## 4. Discussion

Our study showed that septic patients with BSIs from XDR ABCoR have a significantly higher mortality when compared to septic patients with XDR ABCoS BSIs. Interestingly, patients with ABCoR BSIs had a fulminant course, with rapid progress to septic shock and very high sepsis-related mortality, dying in just a few days from the onset of sepsis.

Our patients with AB BSIs had a high overall mortality rate of 64.5%, and colistin resistance itself seemed to impact mortality, as all patient with ABCoR died. This is in accordance with previous studies that reported mortality from AB BSIs ranging from 52.3–84.7% depending on the study population [27,38,39,40,41]. In our study, all the patients with ABCoR BSI eventually died. In the literature we found only three studies demonstrating that ABCoR infection, in general, has a high mortality rate and that colistin resistance is an independent risk factor for mortality [25,42,43]. In a recent retrospective multi-centric study by Aydin et al., where healthcare-associated infections were investigated in 20 tertiary care centers, ABCoR was present in only 2.1% of the cases (*n* = 9 patients). Nevertheless, CoR was significantly associated with increased fatality rate (55,7%) [42]. In the retrospective study by Lertsrisatit et al., among 19 patients with ABCoR isolates related to pneumonia, septicemia of unknown origin, and cholangitis, the 30-day mortality was as high as 70.6% [43]. On the other hand, the single-center retrospective US study of Qureshi et al. reported a 30-day all-cause mortality rate due to ABCoR infection (mostly VAP) of only 30% [25]. However, this study had only 20 patients with ABCoR infection or colonization and lacked a comparison group with colistin-susceptible AB cases.

To our knowledge there are only two studies investigating the impact of either ABCoR infection or bacteremia on the mortality of patients pooled exclusively from an ICU population and our findings are in full agreement with the results of these studies: In the recently published prospective observational study from Mantzarlis et al., in ICU patients who required mechanical ventilation for >48 h during a 36-month period, the mortality rate among 20 patients with ABCoR infection was 85% vs. 39% of 57 patients for the ABCoS group [28]. Additionally, in the retrospective study of Katsiari et al., 16 out of 29 patients (55.1%) of an ICU population with ABCoR BSI died [44].

Our results are in contrast with those of a secondary analysis of a randomized controlled trial of patients with severe infections due to carbapenem-resistant AB, where colistin resistance was associated with a significantly lower 28-day mortality of 42.3% [45]. This different result may be explained by the different patient populations chosen for the conduction of the study (i.e., secondary analysis, fewer patients on mechanical ventilation with higher baseline functional capacity).

Our study also revealed that almost three out of four patients with ABCoR BSI presented with fulminant septic shock with a significantly increased mortality within the first 3 days from its onset. We consider this a very important finding since it is first one to reach to the above conclusion with a statistical significance. There are only two other studies that have addressed this [28,44]. The first study was that of Mantzarlis et al., where patients infected with ABCoR died sooner after the onset of the infection in comparison to patients infected with ABCoS (no statement about statistical significance) [28]. In the study of Katsiari et al., authors divided patients with AB bacteremia into three groups, according to their ICU outcome; those who survived, those that died early (within 48 h, i.e., fulminant sepsis) and those that died later (>48 h) after the onset of bacteremia. In this study, ABCoR was higher in the patient group with fulminant sepsis, though this did not reach statistical significance probably due to the small patient sample size [44]. Authors of both studies assume that the fact that ABCoR patients died sooner in comparison to patients infected with ABCoS may be explained by the virulence of ABCoR strains and perhaps the lack of appropriate therapy.

According to the extensive review by Wong et al., in the case of AB infection, antibiotic resistance itself drives the outcome of the patients [46]. Inappropriate antimicrobial therapy seems to be an independent risk factor for mortality not only in XDR AB bacteremia, as already stated [27,41,47], but also in multidrug-resistant AB or carbapenem-resistant AB bacteremia [16,48,49,50,51,52,53,54,55]. Nevertheless, our study failed to demonstrate any statistically significant difference between the two compared groups, possibly due to the small number of patients.

Virulence is probably an important factor. Although most of the experimental studies report a decreased virulence of ABCoR [56], our study supports the hypothesis that in vitro results do not necessarily correlate with the in vivo outcome [57]. On the other hand, it has been experimentally shown that even ABCoR clinical strains recovered during colistin therapy progressively increase their virulence under oxidative stress [57], and when macrophages-, complement-, or neutrophil-related host defense mechanisms are depleted, even avirulent AB strains trigger a lethal lipopolysaccharide Toll-like receptor 4 (TLR-4) mediated sepsis response [46,58]. We assume that this is in part the case with the majority of our patients exhibiting septic shock: in those patients, AB strains escaped innate immune clearance and initiated a TLR4 mediated sepsis. Moreover, it has already been mentioned that the presence of septic shock is indeed a proven independent risk factor of mortality due to XDR AB bacteremia. This is also the case for carbapenem-resistant AB, as confirmed by a recent systemic review and meta-analysis [48]. Unfortunately, we cannot be sure if infection with ABCoR is per se a mortality factor or which host factors, such as underlying diseases, determined the clinical outcome of our patients.

On the other hand, taking into consideration that the presence of septic shock is an important factor in the outcome of XDR AB BSI, one could speculate that in our patients, once host dysfunction became apparent, shifting the balance against the infection was very difficult despite presumed appropriate antibiotic coverage. In our study, a significant proportion of patients that developed sepsis died in less than 72 h, due to septic shock and MODS. This was irrespective of appropriate antibiotic treatment and almost all of these patients had a high SOFA score 24 h before the onset of sepsis, indicating that there was already significant organ dysfunction in place. Our hypothesis is that once organ dysfunction is established in such infections, the Rubicon is crossed in terms of the outcome and appropriate antibiotic treatment by itself fails to prevent mortality. Katsiari et al. [44], reached a similar conclusion, as patients with fulminant sepsis had more severe organ dysfunction and more resistant pathogens, though they failed to show statistical significance probably due to small patient numbers.

Finally, it must be pointed out that patients with ABCoR sepsis had higher Charlson Comorbidity Index score as well as APACHE II score on admission which could affect the outcome, though there was no difference in SOFA score and SOFA change among the two groups. It could be expected that sicker patients have higher mortality, and that APACHE II and Charlson Comorbidity Index scores are risk factors for multidrug resistant infections.

Our study has some limitations that are noteworthy. The most important of these involves the retrospective observational single-center design, and that risk factors for mortality might have been unequally distributed between the two groups. Unmeasured confounders might have also been in place, which in combination with the relatively small patients’ number, renders difficult to differentiate mortality directly attributable to ABCoR BSI from those as a result of comorbidities. Moreover, identification of AB involved only conventional cultures and no molecular typing was performed to characterize the particular AB strains.

On the other hand, to the best of our knowledge, this is the first cohort study investigating the impact of ABCoR BSI on the mortality of patients pooled exclusively from an ICU population. Colistin resistance is associated with higher comorbidity and mortality. It remains debatable whether the cause for high mortality is due to the fact that the more severely ill patients have a higher chance of acquiring an ABCoR BSI or the bacteremia itself. What is undebatable, though, is the fact that when patients develop sepsis due to this XDR pathogen, they have a very poor survival.

## 5. Conclusions

ICU patients with ABCoR BSI infections have excessive mortality and they often develop a fulminant form of sepsis that rapidly progresses to shock and eventually death.

## Figures and Tables

**Table 1:** Characteristics of the total cohort of ICU patients with XDR-AB infection (total study population *n* = 44).

Patients’ Characteristics	
Age, years (mean ± SD)	65.3 ± 17.6
Sex-males (*n*, %)	29 (65.9%)
APACHE II score (median, min–max)	24 (13–40)
Diabetes mellitus (*n*, %)	10 (22.7%)
Chronic heart failure (*n*, %)	5 (11.3%)
Cardiovascular disease (*n*, %)	8 (18.2%)
Chronic kidney disease (*n*, %)	9 (20.5%)
Cirrhosis (*n*, %)	3 (6.8%)
Chronic corticosteroid intake (*n*, %)	7 (15.9%)
Cancer under therapy (*n*, %)	3 (6.8%)
Immuno-suppressive therapy (*n*, %)	4 (9.1%)
Cause of admission (*n*, %)	
Shock of any kind (*n*, %)	10 (22.7%)
ARF, need for MV (*n*, %)	16 (36.4%)
Coma (*n*, %)	17 (38.6%)
Need for monitoring (*n*, %)	1 (2.3%)

ARF: acute respiratory failure, MV: mechanical ventilation.

**Table 2:** Characteristics of *AB* infection.

Parameter	
Blood culture positive (*n*, %)	31 (70.5%)
Fulfil criteria for sepsis (*n*, %)	39 (88.6%)
SOFA score 24 before (median, min–max)	8 (3–18)
SOFA score on day 0 (median, min–max)	11 (6–20)
Difference in SOFA (median, min–max)	3 (2–10)
Fulfil criteria for septic shock (*n*, %)	29 (65.9%)
Days from admission to sepsis, days (median, mix-max)	21 (0–82)
Days from ICU admission to sepsis, days (median, min-max)	12 (0–55)
Days from admission to positive blood cultures, days (median, mix-max)	20 (3–82)
Days from admission to shock, days (median, mix-max)	22 (0–82)
Duration of shock, days (median, mix-max)	4 (1–14)
Lowest pH on day 0 (median, min-max)	7.14 (6.8–7.44)
Levels of lactate on day 0 (median, min-max)	3.4 (1–14)
PaO_2_/FiO_2_ ratio on day 0, (median, min-max)	213 (41–463)
WBC count on day 0 (median, min-max)	15,210 (900–46,100)
CRP on day 0 (median, min-max)	185 (11–479)
Implications	
ARDS (*n*, %)	15 (34.1%)
AKI (*n*, %)	27 (61.4%)
CRRT (*n*, %	14 (31.8%)
Septic myopathy (*n*, %)	3 (6.8%)
DIC/thrombopenia (*n*, %)	24 (54.5%)
Liver dysfunction (*n*, %)	18 (40.1%)

SOFA score: Sequential Organ Failure Assessment score; ICU: Intensive Care Unit; PaO2/FiO2 ratio: ratio of arterial oxygen partial pressure to fractional inspired oxygen; WBC: White Blood Cell; CRP: C-Reactive Protein; ARDS: Acute Respiratory Distress Syndrome; AKI: Acute Kidney Injury; CRRT: Continuous Renal Replacement Therapy; DIC: Disseminated Intravascular Coagulation.

**Table 3:** Comparison between patients with colistin-resistant and colistin-sensitive *A. baumannii* bloodstream infection.

Parameter	Patients with Colistin-Resistant *A. baumannii* Blood Stream Infection *n* = 13	Patients with Colistin-Susceptible *A. baumannii* Blood Stream Infection *n* = 18	*p*
Age, years (mean ± SD)	67.6 ± 11.5	56.4 ± 19.9	0.07
APACHE II score (median, min–max)	28 (14–40)	18 (13–40)	0.01
Charlson Comorbidity Index score (median, min–max)	5 (3–10)	4 (0–8)	0.044
Diabetes mellitus (*n*, %)	2 (15.4%)	3 (16.7%)	NS
Chronic heart failure (*n*, %)	2 (15.4%)	3 (16.7%)	NS
Chronic kidney disease (*n*, %)	2 (15.4%)	3 (16.7%)	NS
SOFA score 24 h before admission (median, min–max)	9 (3–18)	6 (3–12)	NS
SOFA score on day 0 (median, min–max)	14 (6–20)	11 (7–16)	NS
Difference in SOFA (median, min–max)	2.5 (1–10)	4 (4–4)	NS
Fulfil criteria for septic shock (*n*, %)	13 (100%)	14 (77.8%)	NS
Days from admission to sepsis, days (median, mix–max)	14.5 (0–82)	14 (3–38)	NS
Days from ICU admission to sepsis, days (median, min–max)	10 (0–55)	10.5 (0–31)	NS
Days from admission to positive blood cultures, days (median, mix–max)	14 (5–82)	15 (3–50)	NS
Days from admission to shock, days (median, mix–max)	18 (0–82)	12.5 (0–20)	NS
Duration of shock, days (median, mix–max)	1 (0.5–14)	5.5 (1–14)	0.04
Days from sepsis to death (median, mix–max)	3 (1–25)	4 (2–14)	0.006
Lowest pH on day 0 (median, min–max)	7.03 (6.9–7.33)	7.27 (7.11–7.44)	0.03
Levels of lactate on day 0 (median, min–max)	2.8 (1–14)	1.5 (0.8–8.8)	0.0001
Maximum levels of lactate during hospitalization (median, min–max)	11.4 (1.4–26)	1.7 (1–9.1)	0.0001
PaO_2_/FiO_2_ ratio on day 0, (median, min–max)	198 (41–463)	212 (105–385)	NS
WBC count on day 0 (median, min–max)	14,500 (900–28,200)	14,500 (2,700–46,100)	NS
CRP on day 0 (median, min–max)	86.5 (11–430)	172 (28–451)	NS
Implications			
ARDS (*n*, %)	5 (38.5%)	4 (22.2%)	NS
AKI (*n*, %)	12 (92.3%)	6 (33.3%)	NS
Septic myopathy (*n*, %)	0	2 (11.1)	-
DIC/thrombopenia (*n*, %)	12 (92.3%)	3 (16.7%)	0.02
Liver dysfunction (*n*, %)	8 (61.5%)	5 (27.8%)	NS
Mortality, overall	13 (100%)	7 (50%)	0.001
Sepsis-related mortality	9 (69.2%)	4 (22.2%)	0.003

SOFA score: Sequential Organ Failure Assessment score; PaO_2_/FiO_2_ ratio: ratio of arterial oxygen partial pressure to fractional inspired oxygen; WBC: White Blood Cell; CRP: C-Reactive Protein; ARDS: Acute Respiratory Distress Syndrome; AKI: Acute Kidney Injury; DIC: Disseminated Intravascular Coagulation; NS: not significant.
